# About a Possible Impact of Endodontic Infections by *Fusobacterium nucleatum* or *Porphyromonas gingivalis* on Oral Carcinogenesis: A Literature Overview

**DOI:** 10.3390/ijms25105083

**Published:** 2024-05-07

**Authors:** Luca Ciani, Antonio Libonati, Maria Dri, Silvia Pomella, Vincenzo Campanella, Giovanni Barillari

**Affiliations:** 1 Department of Clinical Sciences and Translational Medicine, University of Rome Tor Vergata, 00133 Rome, Italy; ciani.luca95@gmail.com (L.C.); silvia.pomella@uniroma2.it (S.P.); vincenzo.campanella@uniroma2.it (V.C.); 2 Department of Surgical Sciences, Catholic University of Our Lady of Good Counsel of Tirane, 1001 Tirana, Albania; antlib76@libero.it; 3 Department of Surgical Sciences, University of Rome Tor Vergata, 00133 Rome, Italy; mariampdri6@gmail.com

**Keywords:** endodontic infections, *Fusobacterium nucleatum*, *Porphyromonas gingivalis*, oral premalignant diseases, oral squamous cell carcinoma, endodontic neoplasms, odontogenic tumors

## Abstract

Periodontitis is linked to the onset and progression of oral squamous cell carcinoma (OSCC), an epidemiologically frequent and clinically aggressive malignancy. In this context, *Fusobacterium (F.) nucleatum* and *Porphyromonas (P.) gingivalis*, two bacteria that cause periodontitis, are found in OSCC tissues as well as in oral premalignant lesions, where they exert pro-tumorigenic activities. Since the two bacteria are present also in endodontic diseases, playing a role in their pathogenesis, here we analyze the literature searching for information on the impact that endodontic infection by *P. gingivalis* or *F. nucleatum* could have on cellular and molecular events involved in oral carcinogenesis. Results from the reviewed papers indicate that infection by *P. gingivalis* and/or *F. nucleatum* triggers the production of inflammatory cytokines and growth factors in dental pulp cells or periodontal cells, affecting the survival, proliferation, invasion, and differentiation of OSCC cells. In addition, the two bacteria and the cytokines they induce halt the differentiation and stimulate the proliferation and invasion of stem cells populating the dental pulp or the periodontium. Although most of the literature confutes the possibility that bacteria-induced endodontic inflammatory diseases could impact on oral carcinogenesis, the papers we have analyzed and discussed herein recommend further investigations on this topic.

## 1. Introduction

The proper functioning of the human body is aided by its colonization by saprophytic bacteria synthesizing homeostatic factors (e.g., vitamins) or antagonizing pathogenic microorganisms [1].

Mainly because of its direct connection with the external environment, the oral cavity is one of the areas of the human body richest in bacteria: the latter vary in type and number depending on factors specific to the host individual, such as age, sex, eating habits, and geographical area of origin and/or residence [2]. 

In healthy individuals that keep a good oral hygiene, saprophytic bacteria predominantly populate the oral cavity [3]. Poor oral hygiene, especially when prolonged over time, favors the prevalence of pathogenic bacteria over saprophytes, causing caries, abscesses, gingivitis, and/or periodontitis [3]. 

In particular, periodontitis develops following the formation of a microfilm on the teeth (the so termed dental plaque) in which obligate anaerobic Gram-negative bacteria, such as *Fusobacterium nucleatum* (*F. nucleatum*) and *Porphyromonas gingivalis* (*P. gingivalis*), are frequently found [4]. Using their membrane receptors, both *F. nucleatum* and *P. gingivalis* adhere to the cells of the oral cavity, penetrate them, and, at the same time, interact with each other or with other bacterial species that are concomitantly present therein [4]. Regarding such interactions, it is well established that a pathogenic bacterial strain increases the virulence of another pathogenic strain, and that saprophytic bacteria counter the pathogenic ones [3].

Of importance, periodontitis has been found to be linked to the development and clinical progression of squamous cell carcinoma (SCC), an aggressive malignancy which constitutes over 90% of oral cavity neoplasms (oral SCC, OSCC) [5,6]. This pathogenic association is not surprising, given the well-known pro-tumor effects of chronic inflammation: the latter, in fact, implies the long-lasting production of molecules altering cell survival, growth, differentiation or motility, and concomitantly promoting tissue matrix remodeling [7]. However, while the carcinogenic effects of some families of viruses are widely documented, those of bacteria are poorly defined.

Regarding the OSCC, however, we have some more information. In fact, evidence indicates that both *P. gingivalis* and *F. nucleatum* are endowed with tumorigenic activities which are likely to favor OSCC onset and/or progression [5]. Confirming this, the dysplastic and/or hyperplastic lesions that often precede OSCC development (oral pre-malignant diseases, OPMDs) [8] are densely populated by pathogenic bacteria, including *F. nucleatum* or *P. gingivalis*, and display a reduced number of saprophytic bacteria as compared with healthy oral mucosa [9,10]. In OSCCs, the presence of *F. nucleatum* and *P. gingivalis* becomes even more evident, and its intensity parallels the clinical progression of the disease, positively correlating with the tumor size and lymph node metastases [9,10].

It is noteworthy that *F. nucleatum* and *P. gingivalis* are also present in endodontic diseases such as pulpitis, apical granuloma, and radicular cyst [11,12]. That being so, herein we evaluated whether there is any link between endodontic infections by these two bacteria and oral carcinogenesis.

Specifically, in the present review, we first summarize published data concerning *F. nucleatum* or *P. gingivalis* impact on the onset and on the clinical evolution of OPMDs and OSCC. Then, the literature is examined regarding the effects that infection of dental pulp and/or periodontium by *F. nucleatum* or *P. gingivalis* could have on molecular or cellular events leading to the development or the progression of OSCC, endodontic tumors, and/or odontogenic tumors.

Data were searched for in the PubMed Central electronic database of the National Library of Medicine (National Institutes of Health, Bethesda, Maryland, United States of America). The search and the analysis of the retrieved articles were carried out from November 2022 to January 2024. In total, 90 articles were selected for full text screening, and 64 of them were included in the final study. The other articles discussed and cited in this review served to complete the description of the topics herein considered.

## 2. Activities of *P. gingivalis* and *F. nucleatum* Leading to the Development of OPMDs

When they colonize the oral cavity, *P. gingivalis* and *F. nucleatum* release enzymes that digest both the cells and the extracellular matrix, thus dismantling the tissue [5,6]. This is followed by an intense inflammatory response during which leukocytes are recruited to smash the bacteria and the tissue debris that bacteria have generated [5,6,13].

Amidst the recruited leukocytes are monocytes that, once they reach the inflamed site, differentiate into macrophages [5,6,13]. Of note, *F. nucleatum* binds the toll-like receptors (TLR)-2 and -4 that are expressed on the membrane of macrophages: this binding is followed by the activation of the nuclear factor kappa-B (NF-kB) transcription factor that, in turn, promotes the expression of inflammatory cytokines including interleukin (IL)-6 and tumor necrosis factor (TNF) α by macrophages [14] (Table 1).

Additional pro-inflammatory mediators produced by the leukocytes upon their arrival in the injured oral tissue include IL-1 and IL-8 [5,6,65].

Apart from the leukocytes, the inflamed oral tissue is infiltrated by fibroblasts directed at repairing the damaged tissue [65]. Those fibroblasts synthesize the transforming growth factor (TGF)-β1, while nearby macrophages release the epidermal growth factor (EGF) [66]: both types of cytokines deeply influence the proliferation and differentiation of epithelial cells [66].

It is noteworthy that the pro-inflammatory IL-1, IL-6, IL-8, and TNFα, as well as TGF-β1 and EGF, are almost absent from the healthy oral cavity, are found at low levels in periodontitis, are upregulated in OPMDs, and are expressed at even higher levels in OSCCs [65,66], especially when *F. nucleatum* and/or *P. gingivalis* are present [5,6].

In fact, in the inflamed oral mucosa, not only leukocytes, macrophages, and fibroblasts, but also epithelial cells infected by *F. nucleatum* or *P. gingivalis* produce inflammatory cytokines and growth factors [14,15,16,17,18,19,20,21,22,23,24] (Table 1). Consequently, epithelial cells lining the inflamed, bacteria-infected oral cavity are exposed for prolonged times to cytokines which, particularly when combined with each other, promote epithelial-to-mesenchymal transition (EMT) [65,67].

The latter is a multistep process that occurs during the inflammation that precedes and accompanies the repair of a wounded epithelium [65,67]. EMT implies that epithelial cells lose their peculiar static and polarized phenotype and acquire a motile one which resembles that of mesenchymal cells [65,67]. Such a change allows epithelial cells migration that is required for wound healing [65,67].

Briefly, IL-1, IL-6, IL-8, TNF, EGF, and TGF-β trigger intracellular signaling pathways such as the Wingless-related integration site (Wnt)/β catenin, the phosphoinositide 3-kinase (PI3K)/protein kinase B (AKT), and the mitogen-activated protein kinases (MAPK)/extracellular regulated kinases (ERK) [65,67]. This leads to the activation of transcription factors such as NF-kB, zinc finger snail homolog (SNAI), basic helix–loop–helix twist homolog (TWIST), and zinc finger E-box binding homeobox (ZEB) [65,67,68,69]. These transcription factors, in turn, promote the expression of mesenchymal proteins (e.g., vimentin or neuronal cadherin) and pro-invasive proteolytic enzymes, while repressing the expression of epithelial markers such as epithelial (E)-cadherin [65,67,68,69].

Of note, *P. gingivalis* and *F. nucleatum* can activate SNAI and/or ZEB either directly or by inducing the synthesis of IL-1β, TNF-α, EGF, and/or TGF-β [19,25,26,27] (Table 1).

Specifically, when *P. gingivalis* and *F. nucleatum* make contact with epithelial cells, they trigger the EMT-associated transcriptional activity of SNAI, ZEB, TWIST, and β-catenin, thereby upregulating vimentin and pro-invasive enzymes while concomitantly downregulating E-cadherin [26,70,71,72]. This phenomenon, which is particularly evident when oral epithelial cells are infected with *P. gingivalis* and/or *F. nucleatum* [26], explains why the physiological, reversible EMT arising during the transient inflammation that goes along with the repair of the damaged oral mucosa is exacerbated and becomes stable in chronic bacterial periodontitis [9,72].

In this context, it must be highlighted that the EMT arising in the oral cavity upon the infection by pathogenic bacteria is among the main inducers of OPMDs development [9,72].

Indeed, in the inflamed oral cavity, the trans-differentiation of epithelial cells can be accompanied by their proliferation. Specifically, IL-1 directly triggers the growth of dysplastic oral keratinocytes [73]. At the same time, IL-1 stimulates keratinocytes to synthesize IL-6 and IL-8 [73] that, in turn, intensify the EMT process [74]. Moreover, *F. nucleatum* and *P. gingivalis* induce the production of EGF [19] (Table 1), which sparks both the EMT and the proliferation of oral keratinocytes [75]. In addition, *F. nucleatum* and *P. gingivalis* promote, in oral keratinocytes, the synthesis of factors stimulating cell cycle progression (e.g., the cyclin-dependent kinases), and repress the expression or the activity of growth inhibitors such as p53 [32,33,34,35,36] (Table 1). In doing so, *F. nucleatum* and *P. gingivalis* amplify the proliferative stimulus that EGF exerts on keratinocytes.

Altogether, these molecular and cellular events explain why the presence of *F. nucleatum* and *P. gingivalis* in the oral cavity associates with the development of dysplastic/hyperplastic lesions such as OPMDs [9,10].

## 3. Effects of *P. gingivalis* and *F. nucleatum* Leading to the Onset of OSCC

OSCC results from the malignant transformation of oral keratinocytes: this event is facilitated when the carcinogen is acting on trans-differentiated and/or proliferating keratinocytes, i.e., on an OPMD [76,77].

OSCC is induced mainly by chemical pathogens (e.g., polycyclic hydrocarbons or alcohol), although microbial agents such as the human papilloma viruses certainly contribute to oral carcinogenesis [78]. Also, *P. gingivalis* and *F. nucleatum* are likely to play a direct role in OPMD evolution to OSCC, as they release mutagenic substances (e.g., hydrogen sulfide) into the oral tissue they have colonized [28,29,30,31] (Table 1).

Like all carcinomas, OSCC consists of transformed epithelial cells that display a strong resistance to programmed cell death (apoptosis) [79]. In this regard, it must be highlighted that the intracellular signaling pathways stimulated by *F. nucleatum* and *P. gingivalis* hamper apoptosis promoters such as p53 or Bad [40,44] in oral keratinocytes (Table 1). At the same time, signaling by *F. nucleatum* and *P. gingivalis* upregulates the expression of cell survival factors such as AKT, Bcl-2, heat-shock protein 27, superoxide dismutase 2, baculoviral IAP repeat-containing protein 3, or the signal transducers and activators of transcription (STATs) transcription factors [37,38,39,41,42,43,45,46,80] (Table 1).

STATs are ignited by the Janus-associated kinases (JAKs) [6]. In bacterial-infected and inflamed oral cavity, JAKs can be triggered by *F. nucleatum* and *P. gingivalis* [43,46] or upon the binding of IL-1β, IL-6, IL-8, TNF-α, EGF, or TGF-β to their membrane receptors [81,82,83,84,85,86,87]. In this context, it must be underlined that, while they directly activate the JAKs, *F. nucleatum* and *P. gingivalis* stimulate OSCC cells to produce the abovementioned JAK-activating cytokines [14,15,16,17,18,19,20,21,22,23,24].

STATs actuation results in the induction of the expression of genes that impact not only on cell survival, but also on cell proliferation, motility, and differentiation [88]. All these activities well explain the fact that STATs dysregulation accompanies the onset or progression of a variety of human malignancies [88,89].

Among STAT family members, STAT3 is over-activated in OSCCs, where it strengthens the viability and promotes the proliferation of OSCC cells [82,83,86,90].

Despite this, the establishment and growth of the OSCC can still be hampered by host immune reactions directed against the developing tumor [13]. In fact, the growing OSCC undergoes infiltration by immune cells such as B, T, and NK lymphocytes, dendritic cells, as well as two types of tumor-associated macrophages (TAMs): M1 and M2 [13]. The former has an anti-tumor action as it engulfs tumor cells and processes their antigens by presenting them to the lymphocytes in association with class II major histocompatibility complex (MHC-II) molecules which they display at high levels [13]. In contrast, the M2 macrophages bear low levels of MHC-II, are poorly capable of engulfing the OSCC cells, and produce high amounts of pro-tumor molecules [13]. This explains why a high number of infiltrating M2 macrophages correlates with the poor prognosis of OSCC patients [91].

Of relevance, STAT3, which is triggered by *F. nucleatum* or *P. gingivalis* [37,43,46,80], promotes the synthesis of cytokines in OSCCs that depress the response of cytotoxic T lymphocytes against the carcinoma cells [90].

Of utmost interest for the present review, *P. gingivalis* induces the polarization of TAMS toward the M2 phenotype [13], stimulates the generation of myeloid-derived dendritic suppressor cells from monocytes [56,58], and causes T cells anergy and apoptosis [57] (Table 1). For its part, *F. nucleatum* degrades immunoglobulins [55] and protects tumors from immune cells attack by activating the immune inhibitory receptors TIGIT (T cell immunoreceptor with Ig and ITIM domains) and CEACAM1 (CEA Cell Adhesion Molecule 1) [59,60] (Table 1).

## 4. Effects of *P. gingivalis* and *F. nucleatum* Leading to OSCC Progression

When they escape anti-tumor immune responses, OSCC cells proliferate uncontrollably, thereby infiltrating the tissue in which they have developed and replacing pre-existing normal cells [92,93]. This is by reason that OSCC cells are invasive, that is, they produce proteolytic enzymes capable of degrading the intercellular junctions and both the interstitial and the peritumoral extracellular matrix [94].

Cellular invasiveness is a basic feature of the EMT phenotype [65,67]. In this regard, it must be underscored that, when *F. nucleatum* and *P. gingivalis* penetrate the OSCC cells, they activate ZEB and SNAI, thus directly promoting the pro-invasive EMT phenotype of those cancerous cells [19] (Table 1).

Among the proteolytic enzymes produced by OSCC cells, the matrix metalloproteases (MMPs) play a preponderant role in OSCC invasiveness [94]. In this context, both *F*. *nucleatum* and *P. gingivalis* stimulate MMP activity and OSCC cell invasion (Table 1). Specifically, these bacteria trigger intracellular signaling pathways including Wnt, integrin/FAK, or p38 MAPK that, in turn, lead to MMPs expression and actuation [44,48,49,52,53,54,63]. Additional ways through which *P. gingivalis* and *F. nucleatum* provoke OSCC invasion include by producing sodium butyrate [95], a metabolite that promotes MMPs synthesis [96]. Moreover, *F. nucleatum* can also degrade the extracellular matrix directly, that is, via its bacterial proteases [55], while *P. gingivalis* stimulates the locomotion of the carcinoma cells [51].

In summary, *F. nucleatum* and *P. gingivalis* exasperate EMT in OSCC cells, further reducing the levels of E-cadherin and dramatically increasing those of MMPs [69,72].

The combination of E-cadherin downregulation and MMP overexpression loosens intercellular adhesions, causing the detachment of OSCC cells from the tumor and their centrifugal migration, which will eventually expand the OSCC mass [97].

The enlarging OSCC requires greater amounts of oxygen and nutrients that local vessels are unable to provide: the formation of new blood vessels from pre-existing ones (the so-termed angiogenesis) is, therefore, triggered [76,98,99]. In this context, the GroEL protein of *P. gingivalis* has been reported to promote new vessel formation in vivo [61], while infection by *F. nucleatum* impacts angiogenesis-related genes [62].

In OSCC, STAT3 induces the expression of cytokines that promote angiogenesis, hence supporting the growth of the tumor [90]. Among STAT3-induced angiogenic cytokines are the vascular endothelial growth factor (VEGF) and the fibroblast growth factor (FGF)-2 [100,101]. Definitely, *P. gingivalis* capability of turning on STAT could explain the angiogenic effect of the bacterium. Still in this regard, *F. nucleatum* transiently increases the expression of VEGF and its type 1 receptor by endothelial cells [102].

Moreover, *F. nucleatum* stimulates endothelial cells to produce and release IL-1 and TNFα, hence further increasing the concentration of these inflammatory mediators in the microenvironment [102]. Therefore, as the tumor progresses, EMT-promoting transcription factors are more and more activated, until the carcinoma cells acquire a phenotype that is very similar to that of stem cells (cancer stem cells, CSCs) [103,104,105].

Specifically, CSCs appearance in an OSCC is often preceded by the EMT of the carcinoma cells and/or resident stem cells [103,105]. In agreement with *P. gingivalis* capability of sparking pro-EMT transcription factors, the *P. gingivalis*-infected oral epithelial cells express stem cell markers such as CD44 and CD133 [50] (Table 1). Similar effects have been described for *F. nucleatum* in other types of carcinomas [106]. *F. nucleatum* further inhibits the differentiation of gingival stem cells and triggers events linked to their neoplastic transformation [22,64] (Table 1).

Since they are very invasive and plastic, that is, adaptable to the characteristics of tissues other than that in which they have originated, CSCs are very metastatic [105]. Specifically, CSCs rapidly migrate through the peritumoral matrix, degrade the basement membrane, reach the lymphatic or blood capillaries, and penetrate them [65].

However, when they circulate in the blood or lymph, OSCC cells lack the anchorage to a solid substrate which, analogously to any other adherent cell type, they need to survive [107]. Consequently, circulating OSCC cells may undergo a peculiar, fast-occurring type of apoptosis that is termed “anoikis” [107]. In this regard, the activation of the PI3K/AKT signaling pathway promoted by *P. gingivalis* [39] or *F. nucleatum* [47] makes OSCC cells resistant to anoikis [107], thereby effectively favoring OSCC metastasization.

It is noteworthy that, by virtue of their high resistance to apoptosis, CSCs can survive even ionizing radiation or cytotoxic drugs [108,109]: this renders the OSCCs that are rich in CSCs poorly sensitive to anti-tumor therapies [110].

## 5. Possible Impact of Endodontic Infections by *P. gingivalis* or *F. nucleatum* on OSCCs

While the oral cavity hosts hundreds of different microbial species, the dental pulp is, under physiological conditions, sterile [111]. This is because the pulp is separated and protected from the external environment by the dentin and enamel [111].

Damage to these coatings, resulting from traumatic events or tooth decay, allows oral bacteria to penetrate the dental pulp [112]. There, bacteria find nutrients and oxygen that favor the replication of saprophytic species [111,113,114]. The latter then consume the oxygen and the nutrients and produce catabolites: all this changes the characteristics of the microenvironment in such a way as to favor the prevalence of pathogenic anaerobic bacteria, *P. gingivalis* and *F. nucleatum* included [111,113,114].

**Figure 1:**
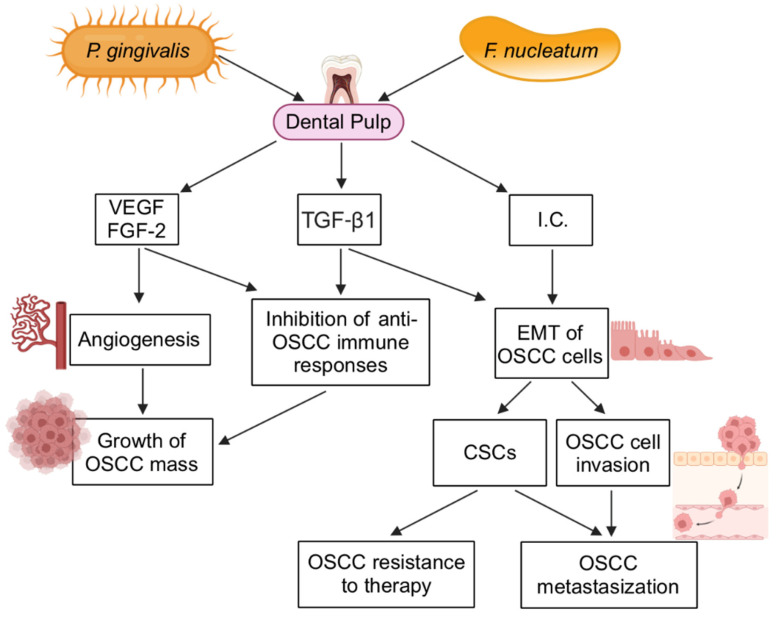
Theoretical effects that dental pulp infection by *P. gingivalis* and/or *F. nucleatum* could have on OSCCs. Arrows symbolize the direction of connections. Abbreviations: CSC, cancer stem cell; EMT, epithelial-to-mesenchymal transition; FGF, fibroblast growth factor; I.C., inflammatory cytokines; TGF, transforming growth factor; VEGF, vascular endothelial growth factor. Created with BioRender.com.

However, the pulp reacts against the invading microorganisms [11]. Specifically, when bacteria arrive in the pulp, cells that are present therein such as the odontoblasts lining the pulp chamber toward the dentine, the dendritic cells, and the fibroblasts produce chemokines that recruit, to the site of the lesion, the leukocytes that will fight the bacteria [11]. As an example of this, when *P. gingivalis* or *F. nucleatum* binds the microbial recognition receptors TLR2 and TLR4 on the surface of odontoblasts, NF-kB is actuated, resulting in odontoblast production of the leukocyte-activating TNFα and IL-8 [115] (Figure 1). Other cytokines whose synthesis by dental pulp cells is triggered by pathogenic bacteria include IL-1β, IL-6, and TGF-β1 [116,117].

In addition to the odontoblasts, the dendritic cells, and the fibroblasts, the dental pulp is populated by stem cells (dental pulp stem cells, DPSCs): these are characterized by a high replicative index and by their capability of differentiating into a wide variety of cell types, which include odontoblasts, osteoblasts, chondrocytes, adipocytes, neural cells, and endothelial cells [118,119,120,121,122]. DPSCs carry out reactive, defensive, and regenerative actions that are mediated by the molecules these stem cells produce [123]. Specifically, DPSCs have been shown to synthesize and release inflammatory mediators such as TNFα and growth factors including VEGF, FGF-2, and TGF-β1 [124].

It is noteworthy that both *P. gingivalis* and *F. nucleatum* are capable of infecting DPSCs where they trigger NF-κB, which, in turn, activates the expression of inflammatory mediators, increasing their local concentrations [117,125,126] (Figure 1). The same effect can result from the bare exposure of the oral stem cells to *P. gingivalis*, without the need for them to be infected by the bacterium [127]. This is because either *P. gingivalis* or its lipopolysaccharides (LPSs) trigger the phosphorylation of ERK and p38 MAPK in DPSCs, this phenomenon being followed by the production of pro-inflammatory cytokines [127]. In this regard, MAPK/ERK activation is also known to lead to VEGF and TGF-1β expression [128,129]. In addition to VEGF and TGF-1β, FGF-2 as well could likely be upregulated in the infected pulp, as found for other inflamed tissues [130,131]. Therefore, the levels of inflammatory cytokines and/or growth factors could get high in a bacteria-infected and inflamed pulp, where they would be simultaneously released by DPSCs, odontoblasts, dendritic cells, fibroblasts, and by the pulp-infiltrating leukocytes. Given the mitogenic, pro-EMT, and pro-invasive effect of the abovementioned cytokines, these findings suggest that an infection of the dental pulp may impact OSCC (Figure 1).

This hypothesis is corroborated by the finding that, upon their release by DPSCs, the VEGF, FGF-2, TNF-α, and TGF-β1 promote the proliferation of OSCC cells [124] (Figure 1). Consistently, other studies have described the positive impact of each of the aforecited molecules on OSCC cell growth [56,132,133]. In addition, the same cytokines could favor OSCC progression also because of their capability of stimulating the invasiveness of carcinoma cells and of maintaining or even exacerbating their dedifferentiation status [132,133,134,135,136] (Figure 1). Moreover, due to their immunosuppressive properties, VEGF, FGF-2, and TGF-β1 could support OSCC growth also in an indirect fashion, that is, by inhibiting the immune response that OSCC-infiltrating leukocytes exert against the cancer cells [137,138,139] (Figure 1). Last but not least, the VEGF and FGF-2 released by DPSCs could enlarge the OSCC mass by promoting angiogenesis [76,98,99] (Figure 1).

Taken together, all these data and considerations strengthen the hypothesis of a pathogenetic link between pulp bacterial infections and OSCC.

In the absence of the proper endodontic therapy, a deep dental caries causes pulp necrosis that, in turn, permits bacteria to replicate in the root canals and reach the alveolar bone through the apical foramen [11,112,140].

Upon bacterial infection of the root canals, inflammation develops at the interface between the infected radicular pulp and the periodontal ligament, eventually resulting in the destruction of periodontal tissues and the resorption of the alveolar bone [112,140]. In this context, it is noteworthy that the inflammatory reaction promoted by *P. gingivalis* inhibits the mineralization capability of cementoblasts [141]. In addition, *P. gingivalis* can directly promote the apoptosis of cementoblasts [142], while its LPSs inhibit cementoblast growth [143].

**Figure 2:**
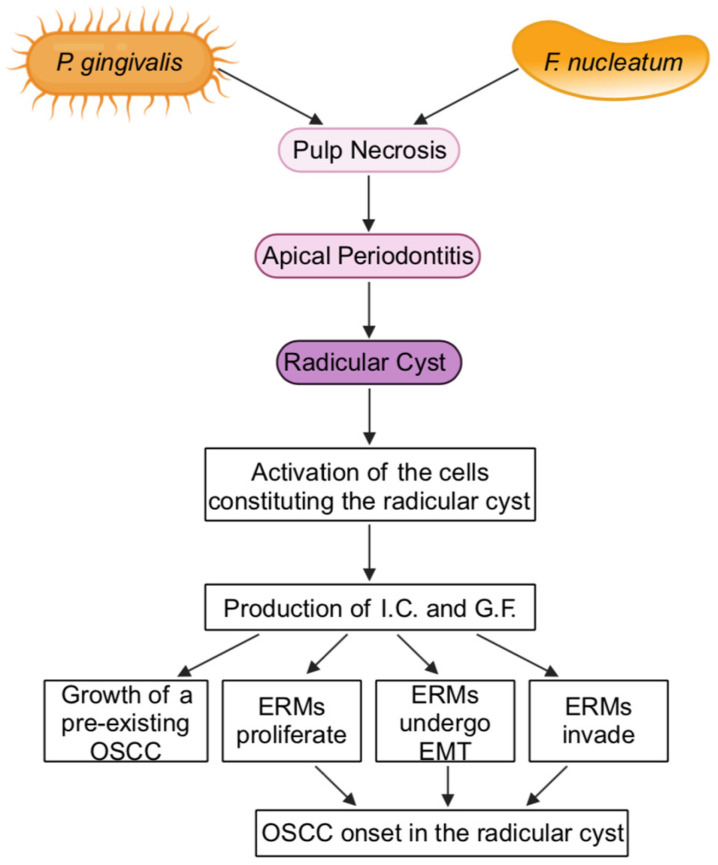
Theoretical effects that an apical periodontitis by *P. gingivalis* and/or *F. nucleatum* could have on OSCCs. Arrows symbolize the directions of connections. Abbreviations: ERMs, epithelial cell rests of Malassez; G.F., growth factors; I.C., inflammatory cytokines. Created with BioRender.com.

Such a disruption of the periodontium defines the “apical periodontitis”, which can lead to the formation of apical granulomas and, subsequently, radicular cysts [11] (Figure 2).

Radicular cysts are lined by a stratified epithelium that derives from the proliferation of the so-called “epithelial cell rests of Malassez” (ERM) residing in the periodontal ligament [144,145]. ERMs are embryonic epithelial remnants that maintain the characteristics of stem cells, being able to differentiate into various cytotypes during periodontal repair [146].

In radicular cysts, ERMs proliferate because they are stimulated by inflammatory cytokines secreted by the various cytotypes that populate the cyst [144,147,148,149,150,151] (Figure 2). Specifically, in addition to epithelial cells, the cyst contains fibroblasts, lymphocytes, monocytes/macrophages, and Langerhans cells [149,150,152,153].

Due to the biosynthetic activity of all those cell types, IL-1β, IL-6, IL-8, and TNFα are expressed in radicular cysts together with growth factors (e.g., TGF-β1, FGF-2, VEGF) or growth factor receptors (e.g., EGF receptor) [151,154,155,156]. In the cysts, MMPs are also detected and are known to be induced by IL-1β, IL-6, IL-8, TNFα, TGF-β1, FGF-2, VEGF, or EGF [147]: these results indicate that inflammatory cytokines and growth factors effectively act on the cells of the cysts, in both an autocrine and paracrine fashion, eventually promoting pro-tumor events such as cell proliferation and invasion (Figure 2).

In most cases, apical lesions heal upon the disinfection and filling of the dental pulp and root canals [112]. In such a contingency, inflammatory cytokines and growth factors are no longer produced or their levels strongly diminish, thus causing the apoptosis of the ERMs that line the radicular cyst [157].

However, sometimes root canals cannot be cleaned and filled in full and this causes infection to abide [112]. In that case, inflammation of the pulp lasts, keeping on IL-1, IL-6, IL-8, and/or TNF production and release [11]. In the eventuality that also the cyst will persist, its cells would prolongedly release these same inflammatory cytokines together with TGF-β1, FGF-2, and VEGF (Figure 2).

In addition to promoting cell growth and invasiveness, these cytokines inhibit cell differentiation or, at the very least, promote EMT, especially when they are all present in the microenvironment at the same time (Figure 2).

Previous work has shown that ERMs can acquire the EMT phenotype [158]. Others have found that DPSCs express genes involved in EMT [159], raising the possibility that DPSCs may also undergo this process.

Given that, the infection of such poorly differentiated cells by pathogenic bacteria could very likely favor the onset of an OSCC (Figure 2). In accordance with this hypothesis, the case of a patient with an infected radicular cyst of the mandible in which OSCC developed has been recently described [160].

## 6. Endodontic Infections by *P. gingivalis* or *F. nucleatum* and Endodontic or Odontogenic Tumors

Despite the simultaneous presence of mitogenic, EMT-inducing, and/or pro-invasive cytokines and that of pro-carcinogenic bacteria, to date no malignant evolution of pulpitis or apical granuloma has been reported in the literature [11,12].

This could depend on several reasons. To begin with, the mesenchymal stromal cells of the dental pulp secrete antibacterial substances [161]. Moreover, cariogenic bacteria produce lactic acid [162], a compound known to exert anti-inflammatory and antitumor actions [163]: this could explain the inverse correlation existing between the presence of tooth decay and the development of head and neck SCC [162]. Furthermore, one should also consider that the onset of a neoplasm in the dental pulp would lead to a reactive pulpitis which sooner or later would be treated as such [12]. In this context, it is likely that the expansion of the tumor mass in the narrow space of the dental chamber would irritate the odontoblasts, inducing them to produce and release new dentin: this would lead to a further shrinking of the dental chamber and, finally, to pulp necrosis [12]. Nonetheless, if one may believe that the small size of the pulp chamber prevents the development of a tumor inside it, this is denied by the finding that cancer metastases can colonize dental pulp [164,165].

Certainly, however, important differences exist between the pathogenetic mechanisms of oral cavity tumors and those of endodontic inflammatory diseases. One of these differences concerns epigenetic alterations such as, for example, those regarding the DNA methylation status [166,167]. Specifically, altered genes in OSCC are those regulating cell survival and proliferation [167], that is, the ones generally most involved in neoplastic induction and progression [166]. In contrast, periodontitis- or pulpitis-associated epigenetic alterations have turned out to mainly affect the genes coding for inflammatory mediators [167]. As such, these results confute, at least in part, the possibility of a neoplastic evolution of an endodontic pathology.

Anyway, regardless of all these considerations, it should be remembered that chronic inflammation can predispose to the development of a neoplasm [7]. It must also be highlighted that the dental pulp is populated by cells that are susceptible to neoplastic transformation such as, for example, the odontoblasts, the fibroblasts, and, above all, the DPSCs [11,168,169]. In this regard, the neoplastic transformation of DPSCs is believed to give origin to the odontogenic myxoma, an invasive and highly recurrent dental tumor [170,171]. Yet, for DPSCs transformation to occur, the intervention of a carcinogen is required [168,169], since these stem cells do not behave like neoplastic cells either in vitro or in vivo [172].

Nevertheless, considering the importance of inflammation and cellular stemness in cancerogenesis [7,76,77,173], it is noteworthy that, upon their release by *P. gingivalis*-infected or *F. nucleatum*-infected DPSCs, the inflammatory cytokines IL-1β, IL-6, and TNFα maintain DPSCs stemness by activating the Wnt/β-catenin signaling [119,120,121] (Table 2). The same effect is also detectable in DPSCs not infected by *P. gingivalis*, but only exposed to the LPSs of the bacterium [118] (Table 2).

*P. gingivalis*, its LPSs, or the inflammatory cytokines induced by this bacterium affect the differentiative potential, but not the survival or growth, of DPSCs [118,119,127]. In contrast, the LPSs of *Escherichia coli*, another bacterium involved in periodontitis [181], increase DPSCs viability [182], possibly favoring endodontic carcinogenesis.

In addition to DPSCs, *F. nucleatum* can also infect the stem cells of the periodontal ligament and apical papilla, thereby halting their differentiation [174] (Table 2). This is due to *F. nucleatum* ability to downregulate the expression of WDR5, a promoter of histone methylation [174]. Still in the stem cells of the periodontal ligament and apical papilla, *F. nucleatum* upregulates the expression of TBX3 and NFIL3, two transcription factors that are involved in embryonic development and are overexpressed in a wide variety of cancers where they functionally hamper cell growth inhibitors, repress cell differentiation, and stimulate cell invasion [174].

As observed in DPSCs, also in the stem cells of the periodontal ligament and of the apical papilla, the LPSs of *P. gingivalis* spark Wnt/β-catenin or p38 MAPK signaling and activate NF-kB, thus triggering the expression of inflammatory cytokines [175,176] (Table 2). Similarly, *F. nucleatum* promotes the synthesis of IL-8 and IL-10 by apical papilla stem cells [177] (Table 2).

Altogether, because of their impact on cellular stemness, these activities of *F. nucleatum* and *P. gingivalis* may have relevance for the onset of dental tumors.

Concerning the effects that *P. gingivalis* exerts on other types of dental cells, results from a previous study indicate that the bacterium triggers both the PI3K/AKT and the MAPK signaling pathways in cementoblasts, thus inhibiting the differentiation of these cells [142] (Table 2).

Others have shown that the LPSs of *P. gingivalis* upregulate IL-1 and MMP-2 expression in cementoblasts [143] (Table 2). This could be of importance for dental tumorigenesis, as IL-1β inhibits DPSCs differentiation [120] and MMP-2 mediates SCs invasion [178] and it is overexpressed in odontogenic neoplasms, contributing to their invasive behavior [171].

Once again regarding a possible role for pathogenic bacteria in odontogenic tumors, evidence suggests a link between endodontic infections and ameloblastoma, the most common odontogenic tumor [183]. In this regard, it is useful to remind that, although it is benign, ameloblastoma can rapidly grow and be locally invasive, thereby causing significant morbidity [183]. 

Of note, the odontogenic ameloblast-associated protein (ODAM) is diffusely and highly expressed in the *P. gingivalis*-infected sub-gingival regions, as compared to the uninfected ones [180] (Table 2). In physiologic conditions, ODAM is expressed at low levels, mediating the adhesion of the junctional epithelium to the tooth surface [184]. As for pathologic settings, ODAM is overexpressed in ameloblastoma as well as in a variety of carcinomas such as that of the stomach, lung, or mammary gland [179].

Furthermore, although it is not known whether *F. nucleatum* or *P. gingivalis* can infect the ameloblasts, the same molecules that are upregulated or activated in OSCC cells infected with *F. nucleatum* or *P. gingivalis* (e.g., ZEB, SNAI, TWIST, β-catenin, the MMPs, IL-8, and vimentin) are also overexpressed in ameloblastoma [185,186,187,188].

## 7. Conclusions and Future Directions

Results from clinical–epidemiological studies indicate that OSCC development and progression are favored by bacterial periodontitis [5,6]. In this context, *P. gingivalis* and *F. nucleatum*, two causative agents of periodontitis, colonize OPMDs and OSCCs [9,10] where they exert tumorigenic activities. In particular, *P. gingivalis* and *F. nucleatum* support the onset of OPMDs and their progression to OSCCs by promoting the transdifferentiation, survival, proliferation, and invasiveness of oral epithelial cells [19,25,26,27,28,29,30,31,32,33,34,35,36,37,38,39,40,41,42,43,44,45,46,47,48,49,50,51,52,53,54,55,63,64]. In addition, *P. gingivalis* and *F. nucleatum* actions can sustain the growth and metastatization of an established OSCC [13,55,56,57,58,59,60,61,62]. All these effects of *P. gingivalis* and *F. nucleatum* are directly promoted by the two bacteria and/or are mediated by the cytokines they induce [14,15,16,17,18,19,20,21,22,23,24].

Given that *P. gingivalis* and *F. nucleatum* are also present in pulpitis, apical granulomas, or radicular cysts [11,12], here we discuss the literature concerning any eventual pathogenetic link between endodontic infections by the abovecited bacteria and oral carcinogenesis.

Specifically, here we report that *P. gingivalis* and/or *F. nucleatum* spark, in dental pulp cells or periodontal cells, the synthesis of cytokines that, in turn, trigger the survival, growth, and invasion of OSCC cells [115,116,117,124,125,126,127,132,133,134,135,136]. Furthermore, the two bacteria and the cytokines induced by them stop the differentiation of DPSCs and periodontal stem cells while concomitantly stimulating their growth and invasiveness [118,119,120,121,174,175,176,177] and releasing mutagenic substances [28,29,30,31].

In conclusion, the set of data discussed here, recovered from the fragmented literature produced on this specific topic, lead to further investigation into the effects that a bacterial endodontic infection could have on oral carcinogenesis.

Noteworthy is the fact that many of the pro-tumor activities of *P. gingivalis* and *F. nucleatum* depend on the capability that the two bacteria have to activate the PI3K/AKT intracellular signaling pathway that is key to OSCC development and progression [39,47,189].

Since several human malignancies display PI3K/AKT dysregulation, antagonists of this pathway have been developed that exert anticancer activity [190]. As for OSCC, PI3K/AKT inhibitors hamper the proliferation and invasion of the carcinoma cells and increase their sensitivity to conventional antitumor therapies [191,192,193,194].

However, regarding a clinical use of PI3K/AKT inhibitors against OSCC, one should consider that, in this tumor, AKT is continuously reactivated due to the peculiar cellular and molecular characteristics of the tumor microenvironment [189]. This has suggested the usage of combinatorial therapeutic regimens directed against OSCC. Consistently, treatment of OSCC patients with PI3K/AKT inhibitors combined with cytostatic/cytotoxic chemotherapeutics has provided promising results [189,190].

Based on the data herein described, OSCC patients could take conventional chemotherapeutics and PI3K/AKT inhibitors together with antibiotics and anti-inflammatory drugs. Because the oral cavity is accessed effortlessly, anti-OSCC drugs would be administered also topically, thus minimizing their collateral effects.

## Figures and Tables

**Table 1:** Direct pro-tumor effects of *P. gingivalis* and *F. nucleatum*.

Pro-Tumor Effect	*P. gingivalis*	*F. nucleatum*
Triggering of the synthesis of inflammatory cytokines or growth factors by infected cells	Wang Q et al. [15]; Ramage G et al. [16]; Milward MR et al. [17]; Yee M et al. [18]; Abdulkareem AA et al. [19]	Park SR et al. [14]; Abdulkareem AA et al. [19]; Aral K et al. [20]; Hung SC et al. [21]; Kang W et al. [22]; Kang W et al. [23]; Kurgan S et al. [24]
EMT induction	Abdulkareem AA et al. [19]; Sztukowska MN et al. [25]; Lee J et al. [26]; Abdulkareem AA et al. [27]	Abdulkareem AA et al. [19]; Abdulkareem AA et al. [27]
Release of mutagenic substances	Nguyen LH et al. [28]	Ma Z et al. [29]; Vital M et al. [30]; Zhang S et al. [31]
Stimulation of cell proliferation	Kuboniwa M et al. [32]; Chang C et al. [33]; Pan C et al. [34]	Geng F et al. [35]; Binder Gallimidi A et al. [36]
Upregulation of cell survival factors	Gao S et al. [37]; Lee J et al. [38]; Yilmaz O et al. [39]; Yao L et al. [40]; Nakhjiri SF et al. [41]; Hoppe T et al. [42]; Li J et al. [43]	Da J et al. [44]; Rath-Deschner B et al. [45]; Duan C et al. [46]
Induction/enhancement of cell invasion	Shen S et al. [47]; Inaba H et al. [48]; Inaba H et al. [49]; Ha NH et al. 2016 [50]; Meng F et al. [51]	Da J et al. [44]; Harrandah AM et al. [52]; Kamarajan P et al. [53]; Uitto VJ et [54]; Bachrach G et al. [55]
Impairment of anti-tumor immunity	Liu S et al. [13]; Arjunan P et al. [56]; Groeger S et al. [57]; Wen L et al. [58]	Bachrach G et al. [55]; Gur C et al. [59]; Gur C et al. [60]
Triggering of tumor angiogenesis	Lin FY et al. [61]	Li Z et al. [62]
Promotion of cellular stemness	Ha NH et al. [63]	Kang W et al. [23]; Kang W et al. [64]

**Table 2:** *P. gingivalis* or *F. nucleatum* effects possibly affecting dental tumorigenesis.

Event	Effect	References
Upon their infection by (or exposure to) Fn or Pg, the dental pulp SCs release IC	IC inhibit the differentiation of dental pulp SCs	Rothermund K et al. [118]; Qin Z et al. [119]; Sonmez Kaplan S et al. [120]; Li M et al. [121];
Upon their infection by (or exposure to) Fn or Pg, the SCs of the periodontal ligament or apical papilla release IC	IC inhibit the differentiation of periodontal ligament or apical papilla SCs	Razghonova Y et al. [174]; Diomede F et al. [175]; Wang J et al. [176]; Rakhimonova O et al. [177]
Fn and Pg upregulate both IL-1 and MMP-2 expression by cementoblasts	IL-1 inhibits the differentiation of SCs and cementoblasts. MMP-2 mediates cementoblasts invasion	Sonmez Kaplan S et al. [120]; Ma L et al. [142]; Bozkurt SB et al. [143]; Miyagi 2012 [171]; Neth P et al. [178]
Pg upregulates the expression of the ODAM-associated protein	Possible impact on the onset and/or progression of ameloblastomas	Kestler DP et al. [179]; Nakayama Y et al. [180]

Abbreviations: IC, inflammatory cytokines; IL, interleukin; Fn, *Fusobacterium nucleatum*; MMP, matrix metalloproteinase; ODAM, odontogenic ameloblast; Pg, *Porphyromonas gingivalis*; SCs, stem cells.
